# Supplementary material for: Postmortem high-dimensional immune profiling of severe COVID-19 patients reveals distinct patterns of immunosuppression and immunoactivation
Source: Nat Commun. 2022 Jan 12;13:269. doi: 10.1038/s41467-021-27723-5 (PMC8755743; doi:10.1038/s41467-021-27723-5)
Supplement: Supplementary file 1 — Supplementary Information [file 41467_2021_27723_MOESM1_ESM.pdf]

Supplementary Information

Postmortem high-dimensional immune profiling of severe COVID-19 patients reveals distinct patterns of immunosuppression and immunoactivation

Haibo Wu[#1], Peiqi He[#2,3,4], Yong Ren[#5], Cheng Sun[*2,3,4]

[1] Department of Pathology, the First Affiliated Hospital of USTC, Division of Life Sciences and Medicine, University of Science and Technology of China, Hefei, Anhui, China, 230036

[2] Hefei National Laboratory for Physical Sciences at Microscale, the CAS Key Laboratory of Innate Immunity and Chronic Disease, School of Basic Medical Sciences, Division of Life Sciences and Medicine, University of Science and Technology of China, Hefei, 230027, China

[3] Institute of Immunology, University of Science and Technology of China, Hefei, 230027, China

[4] Transplant & Immunology Laboratory, the First Affiliated Hospital of USTC, Division of Life Sciences and Medicine, University of Science and Technology of China, Hefei, Anhui, 230001, China

[5] Department of Pathology, the First Hospital Affiliated to Army Medical University, Chongqing 400038, China

# These authors contributed equally to this work.

* Corresponding author: charless@ustc.edu.cn (C.S.).

Supplementary figure 1

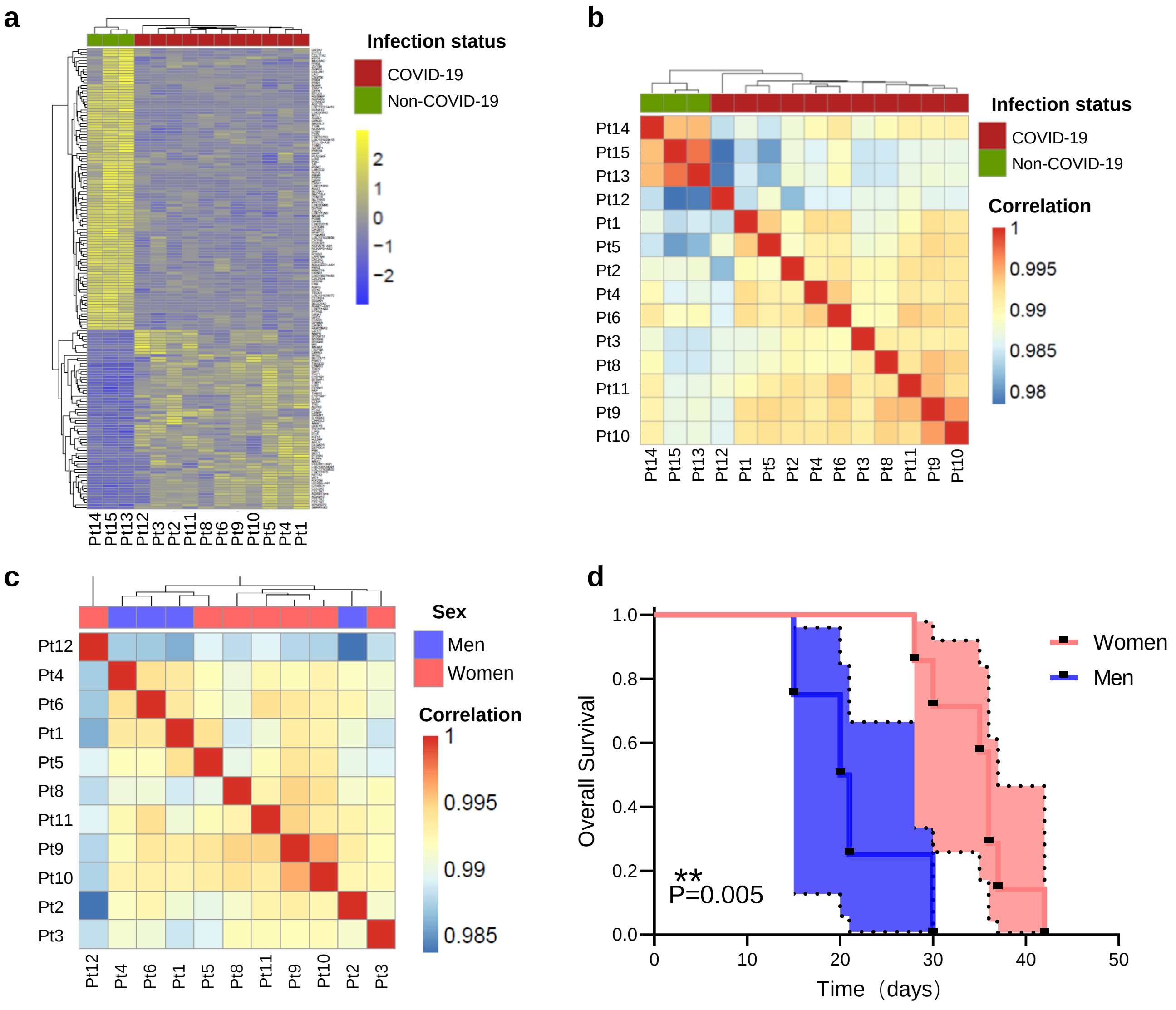

Supplementary Figure 1: Bulk RNA-sequencing differentiates COVID-19 decedents from non-COVID-19 decedents and men from women. (**a**) Heatmap showing the differential gene expression in the lung tissue of COVID-19 (n=11) and non-COVID-19 (n=3) patients. (**b**) Hierarchical clustering of decedents in (**a**) based on bulk RNA-sequencing of lung tissue. (**c**) Hierarchical clustering of men (n=4) and women (n=7) with COVID-19 based on bulk RNA-sequencing of lung tissue. (**d**) Kaplan-Meier survival curve of men (n=4) and women (n=7) with COVID-19. *p*-value: two-sided log-rank test.

Supplementary figure 2

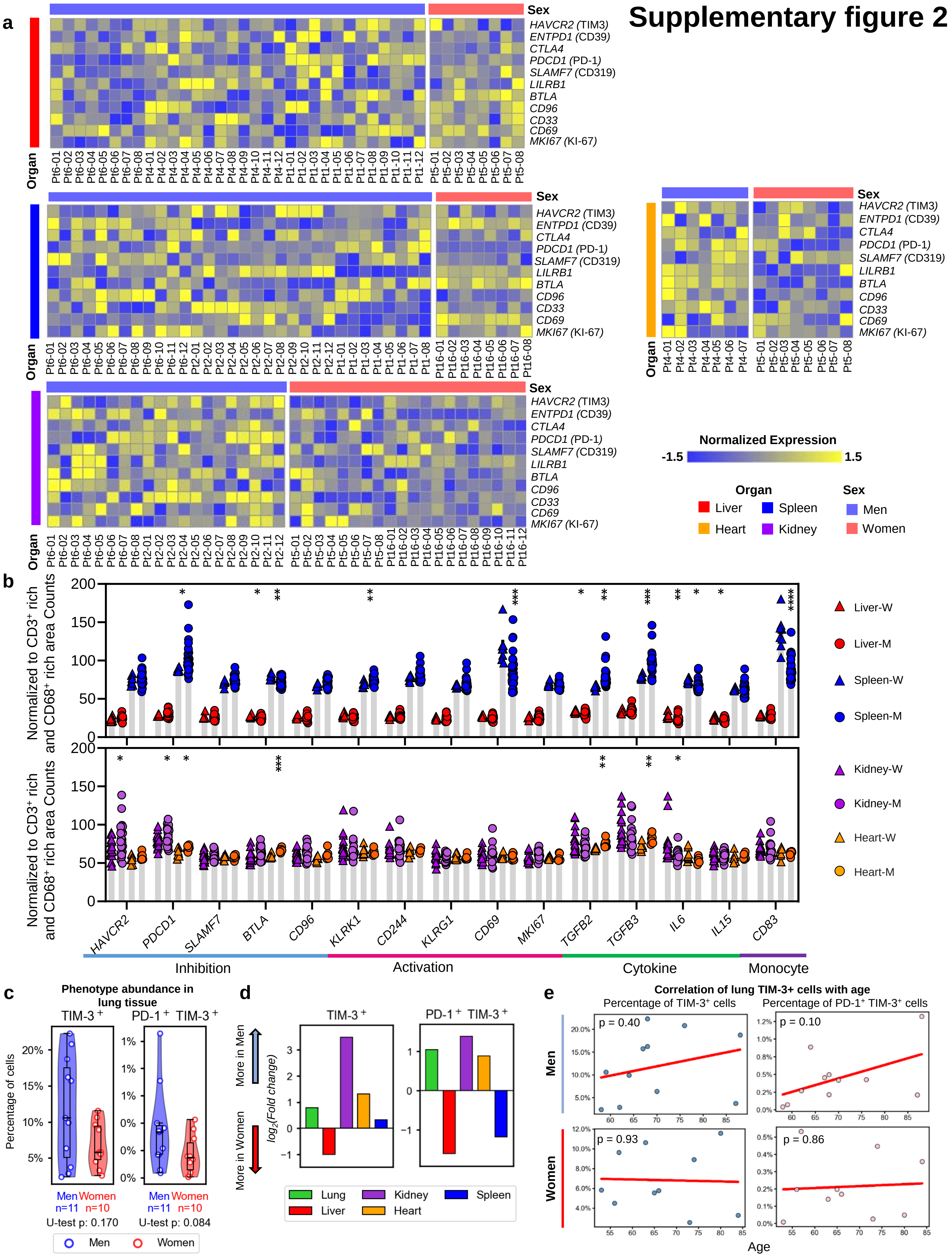

Supplementary Figure 2: TIM-3- and PD-1-mediated immunosuppression is more pronounced in men than in women with COVID-19. (**a**) Heatmap showing the differential gene expression between men (n=4) and women (n=2) from liver (red, 40 ROIs from 4 decedents), heart (orange, 15 ROIs from 2 decedents), spleen (blue, 40 ROIs from 4 decedents) and kidney (purple, 40 ROIs from 4 decedents). (**b**) Comparison of selected genes expression between sexes from liver (red, 40 ROIs from 4 decedents), heart (yellow, 15 ROIs from 2 decedents), spleen (blue, 40 ROIs from 4 decedents) and kidney (purple, 40 ROIs from 4 decedents). Each point represents one ROI. Colored dots represent different samples, liver (red, Liver-M = 32, Liver-W = 8), spleen (blue, Spleen-M = 32, Spleen-W = 8), kidney (purple, Kidney-M = 20, Kidney-W = 20) and heart (yellow, Heart-M = 7, Heart-W = 8). Data are shown as mean ± s.e.m. *p*-liver(*BTLA*) = 0.02; *p*-liver(*TGFB2*) = 0.03; *p*-liver(*IL6*) = 0.002; *p*-liver(*IL15*) = 0.02; *p*-spleen(*PDCD1*) = 0.04; *p*-spleen(*BTLA*) = 0.002; *p*-spleen(*KLRK1*) = 0.006; *p*-spleen(*CD69*) = 0.0007; *p*-spleen(*TGFB2*) = 0.004; *p*-spleen(*TGFB3*) = 0.0008; *p*-spleen(*IL6*) = 0.02; *p*-spleen(*CD83*) < 0.000001; *p*-kidney(*HAVCR2*) = 0.04; *p*-kidney(*PDCD1*) = 0.045; *p*-kidney(*IL6*) = 0.01; *p*-heart(*PDCD1*) = 0.01; *p*-heart(*BTLA*) = 0.0002; *p*-heart(*TGFB2*) = 0.005; *p*-heart(*TGFB3*) = 0.002; Unpaired two-tailed Student's *t*-tests, $*p < 0.05$, $**p < 0.01$, $***p < 0.001$, $****p < 0.0001$. (**c**) Comparison of percentage of TIM-3$^+$ (left) or PD-1$^+$TIM-3$^+$ (right) cells in lung tissue between men (n=11) and women (n=10). *p*-values: two-tailed Student's t-test. (**d**) Differences in percentages of TIM-3$^+$ cells (left) or PD-1$^+$TIM-3$^+$ cells (right) between men and women in various organs. (**e**) Correlation between age and percentage of TIM-3$^+$ cells or TIM-3$^+$PD-1$^+$ cells in lung tissue of men (top) and women (bottom). *p*-values: two-tailed Wald test.

Supplementary figure 3

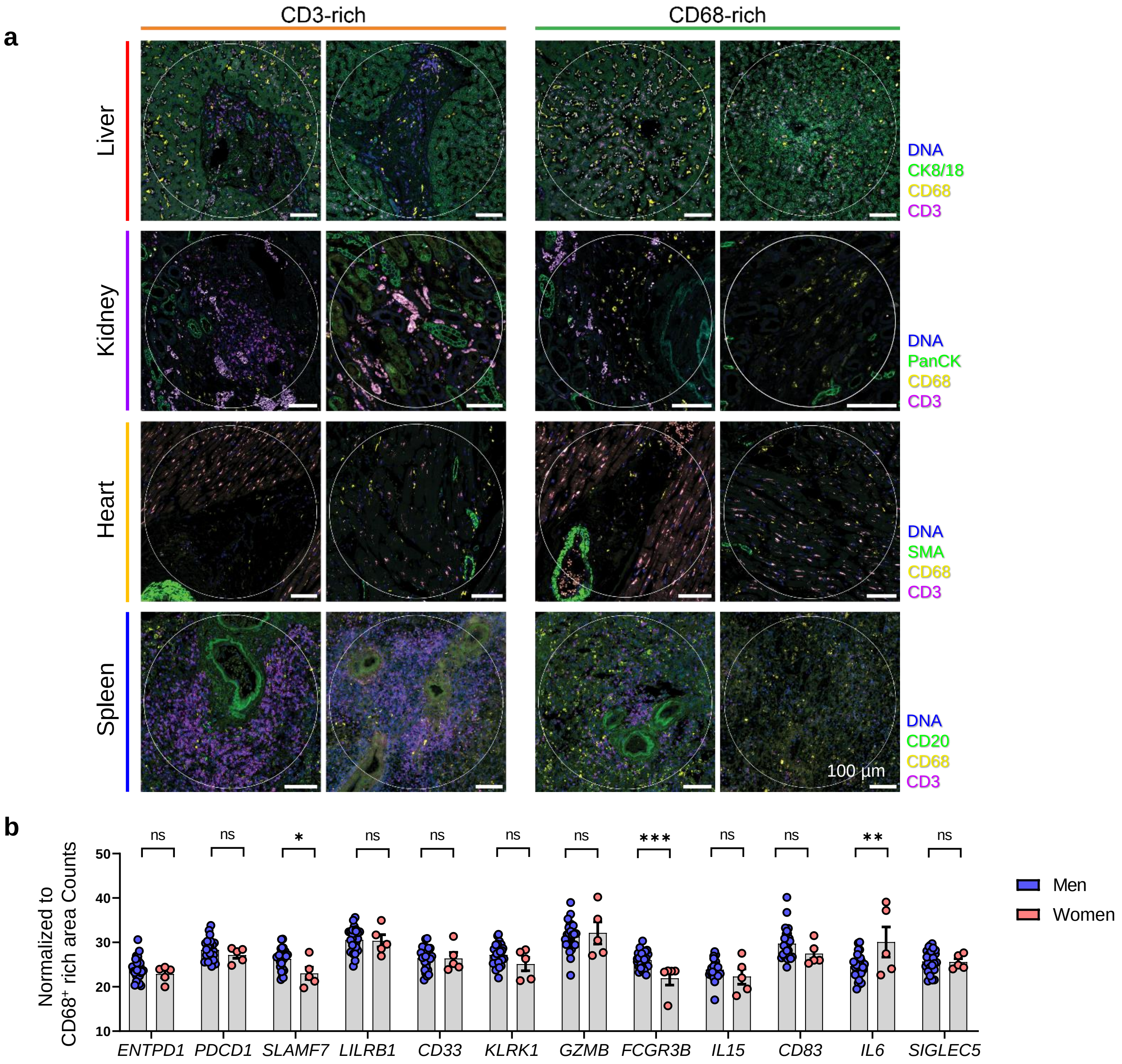

Supplementary Figure 3: **Immune marker expression differs between CD3⁺-rich and CD3⁺-poor regions.** (**a**) Left: Representative CD3-rich DSP ROIs of various organs (Liver n =20, Kidney n=30, Heart n=6, Spleen n=21). Right: Representative CD3-rich DSP ROIs of various organs (Liver n=20, Kidney n=10, Heart n=9, Spleen n=19). (**b**) Comparison of selected genes expression in CD68⁺ rich regions between sexes from lung tissues. Each point represents one ROI. Colored dots represent different sex, Women(red, n = 5) , Men(blue, n = 26). Data are shown as mean ± s.e.m. *p*(*SLAMF7*) = 0.01; *p*(*FCGR3B*) = 0.0007; *p*(*IL6*) = 0.007. Unpaired two-tailed Student's *t*-tests, *$p < 0.05$, **$p < 0.01$, ***$p < 0.001$, ****$p < 0.0001$, ns: not significant.

Supplementary figure 4

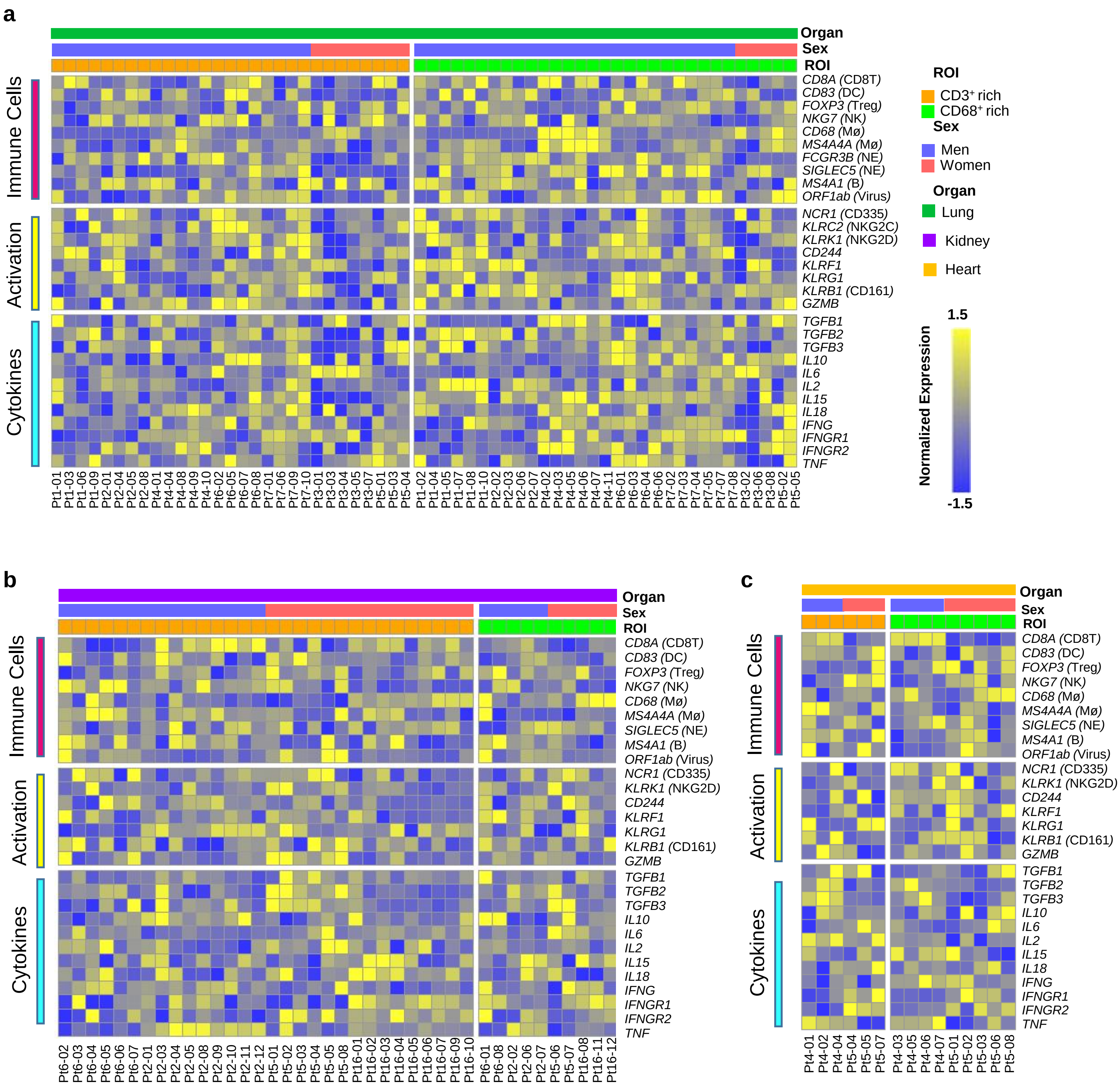

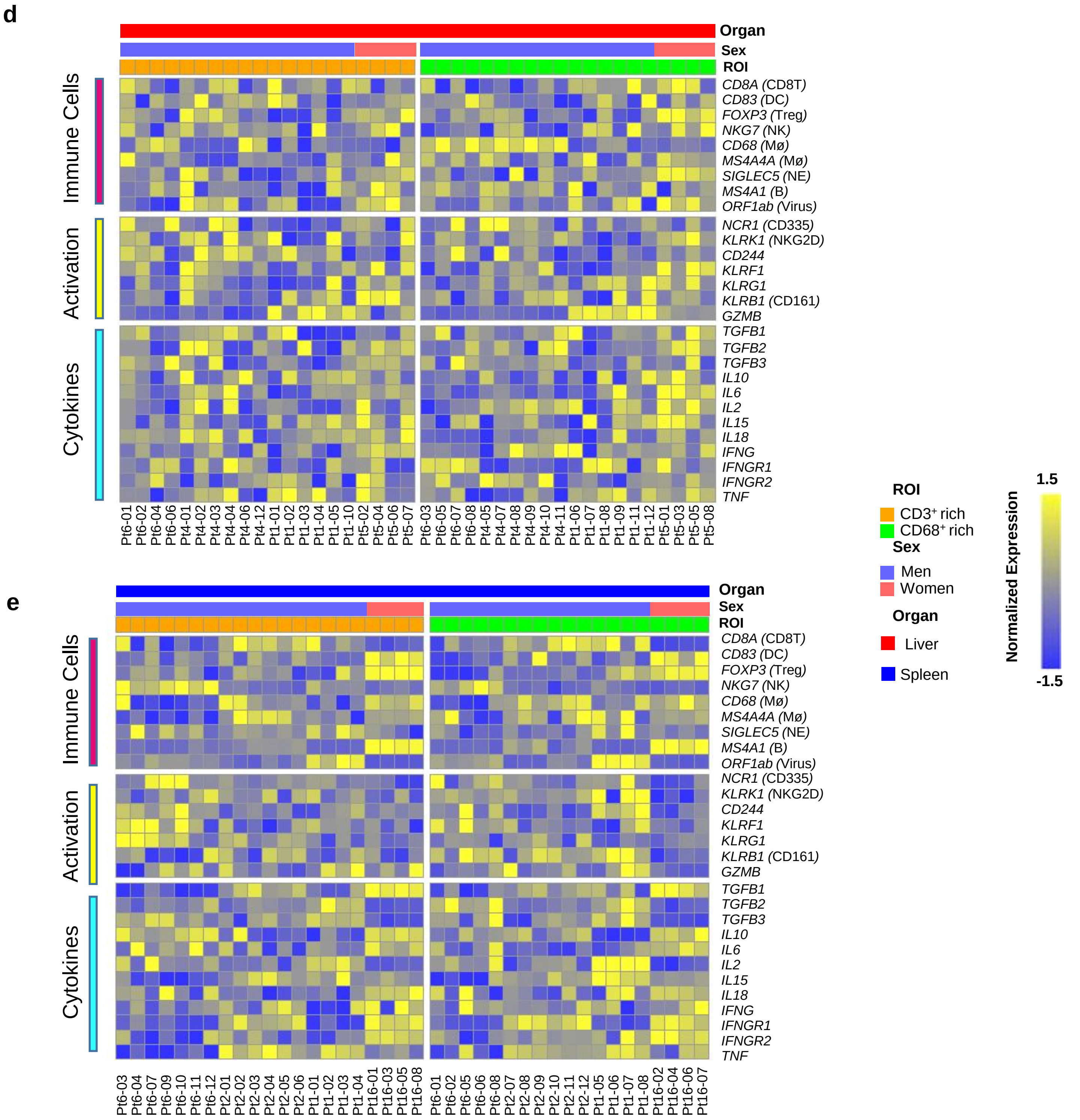

Supplementary Figure 4: **The differences of immune gene profiles between CD3⁺-rich and CD68⁺-rich regions in multi-organs.** (**a-e**) Normalized gene expression of immune cell markers, activation markers, and cytokines between men and women in CD3-rich and CD68-rich ROIs from lung (**a**, 60 ROIs from 7 decedents, men= 2, women = 5), kidney (**b**, 40 ROIs from 4 decedents, men= 2, women = 2), heart (**c**, 15 ROIs from 2 decedents, men= 1, women = 1), liver (**d**, 40 ROIs from 4 decedents, men= 3, women = 1). spleen (**e**, 40 ROIs from 4 decedents, men= 3, women = 1). Z -scale from -1.5 to 1.5.

Supplementary figure 5

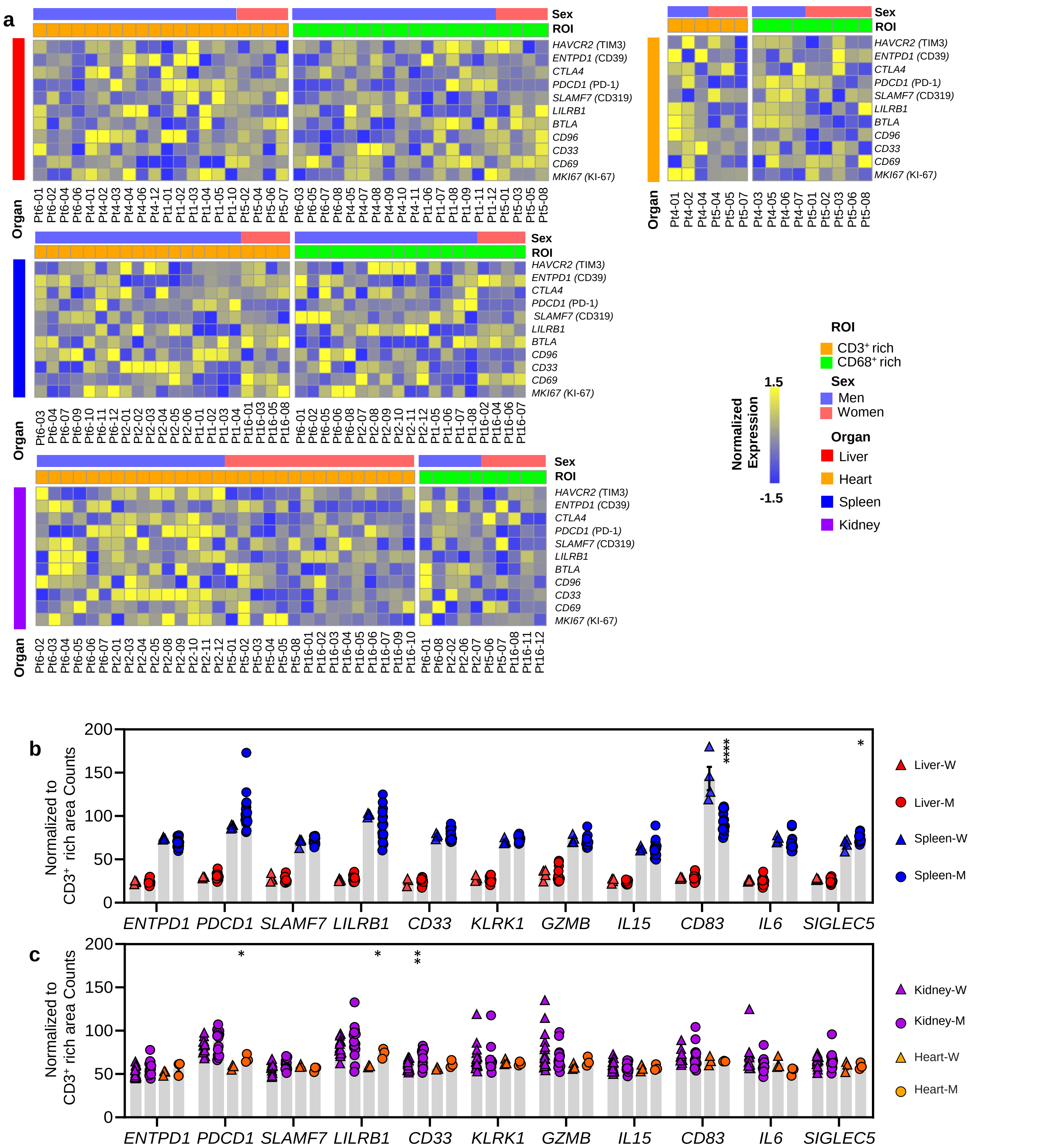

Supplementary figure 5

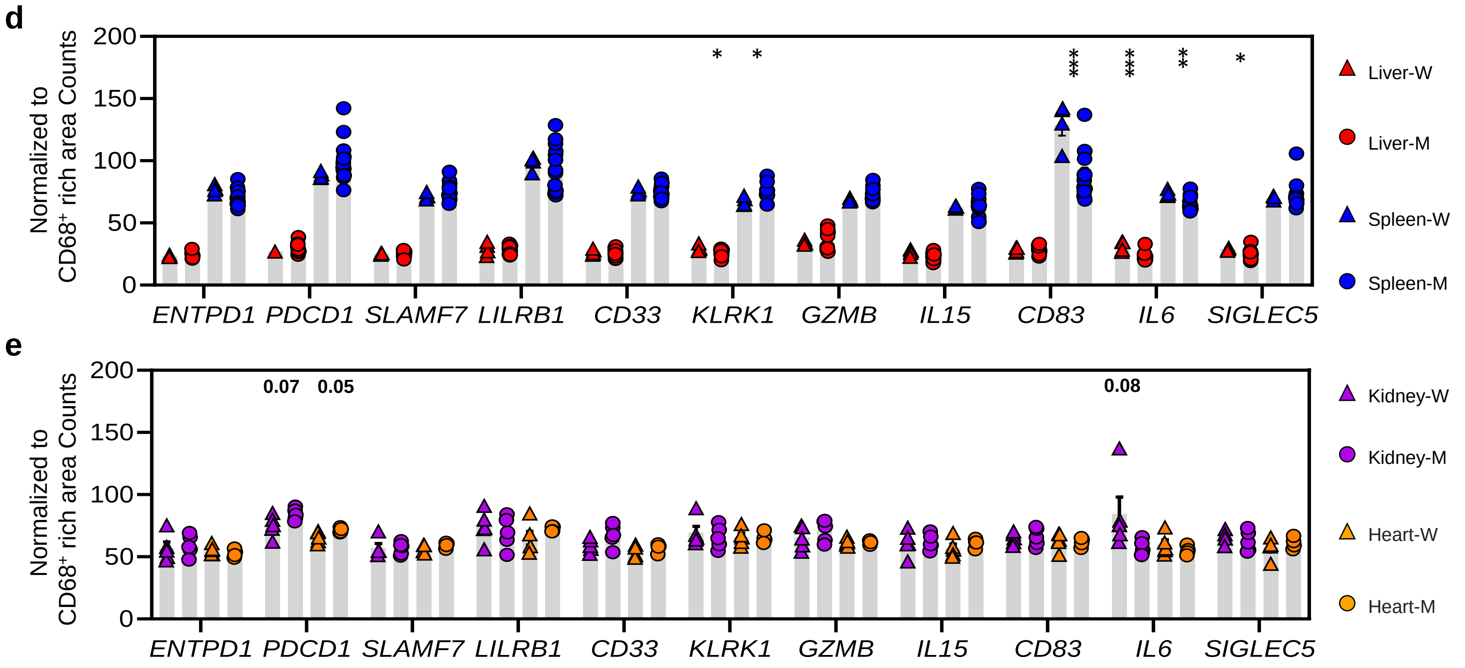

Supplementary Figure 5: **The differences of gene expression between $CD3^+$-rich and $CD68^+$-rich regions in multi-organs.** (**a**) Normalized gene expression of immunosuppression-related genes in $CD3^+$ rich and $CD68^+$ rich from liver (red, 40 ROIs from 4 decedents), heart (orange, 15 ROIs from 2 decedents), spleen (blue, 40 ROIs from 4 decedents) and kidney (purple, 40 ROIs from 4 decedents). Z - scale from -1.5 to 1.5. (**b-e**) Each point represents one ROI. Colored dots represent different samples. Different shapes represent different sex. Data are shown as mean ± s.e.m. (**b-c**) Comparison of selected genes expression in $CD3^+$ rich regions between sexes in multi-organs. (**b**) liver (red, Liver-M = 16, Liver-W = 4), spleen (blue, Spleen-M = 17, Spleen-W = 4), *p*-spleen(*CD83*) = 0.000009; *p*-spleen(*SIGLEC5*) = 0.04; (**c**) kidney (purple, Kidney-M = 15, Kidney-W = 15) and heart (yellow, Heart-M = 3, Heart-W = 3), *p*-kidney(*CD33*) = 0.007; *p*-heart(*PDCD1*) = 0.04; *p*-heart(*LILRB1*) = 0.01. (**d-e**) Comparison of selected genes expression in $CD68^+$ rich regions between sexes in multi-organs.(**d**) liver (red, Liver-M = 16, Liver-W = 4), spleen (blue, Spleen-M = 15, Spleen-W = 4), *p*-liver(*KLRK1*) = 0.02; *p*-liver(*IL6*) = 0.0003; *p*-liver(*SIGLEC5*) = 0.04; *p*-spleen(*KLRK1*) = 0.02; *p*-spleen(*CD83*) = 0.0009; *p*-spleen(*IL6*) = 0.006; (**e**) kidney (purple, Kidney-M = 5, Kidney-W = 5) and heart (yellow, Heart-M = 4, Heart-W = 5). Unpaired two-tailed Student's *t*-tests, *$p < 0.05$, **$p < 0.01$, ***$p < 0.001$, ****$p < 0.0001$.

Supplementary figure 6

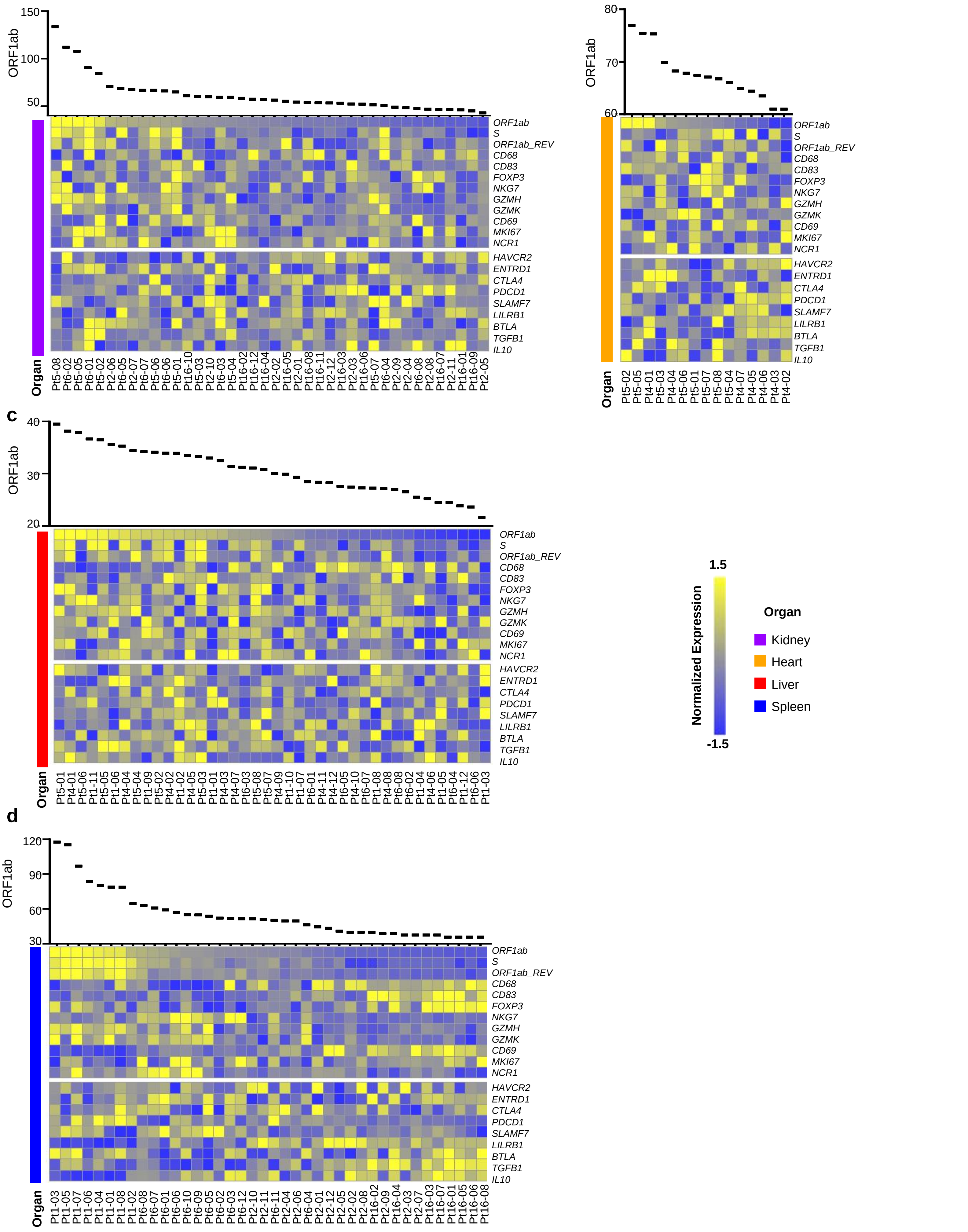

Supplementary Figure 6: Expression of immune-related genes across multiple organs affected by COVID-19. (**a-d**) Heatmap show normalized gene expression level of immune-related genes in ROIs of multi-organs with COVID-19 arranged according to SARS-CoV-2 (*ORF1ab*) expression level. ROIs of kidney (**a**, n=40; from n=4 decedents), heart (**b**, n=15; from n=2 decedents), liver (**c**, n=40; from n=4 decedents) and spleen (**d**, n=40; from n=4 decedents).

Supplementary Table 1. Overview of large-scale autopsy reports in COVID-19 involving IHC

Author	Country	n	Tissues	Techniques used	IHC markers	Virus detection method
Single organ system						
(Su et al., 2020)	China	26	Kidneys	H&E, IHC, TEM	CD235a, CD61, CD31, ACE2, IgG, IgM,	IHC staining for SARS-CoV-NP, TEM
(Carsana et al., 2020)	Italy	38	Lungs	H&E, IHC, TEM	CD45, CD68, CD61, TTF1, p40, Ki67, Masson Trichome	TEM
Multiple organ systems						
(Hanley et al., 2020)	UK	10	Lung, Heart, Brain, Pancreas, Liver, Kidney, Spleen, Lymph nodes, Bone marrow	H&E, IHC, TEM	CD4, CD4, CD8, CD10, CD20, CD34, CD56, CD57, CD61, CD68, CD138, CMV, EBER, FOXP3, Glycophorin C, Granzyme B, Cam5.2, IgD, IgG, IgM, Kappa, Ki-67, Lambda	RT-PCR
(Menter et al., 2020)	Switzerland	21	Lungs, Liver, Heart, Brain, Spleen, Kidneys, Pancreas, Small intestine, Large intestine,	H&E, IHC, TEM	Fibrin, Transthyretin (ATTR), CD3, CD4, CD8, CD20, CD68, MUM1, TTF1	RT-PCR
(Elsoukkary et al., 2020)	USA	32	Lung, Heart, Liver, Spleen, Lymph nodes, Bone marrow, Kidney	H&E, IHC	CD3, CD20, CD61, CD163, C5b-9	IHC staining for SARS-CoV Spike protein, RT-PCR
(This study)	China	22	Lungs, Liver, Heart, Kidney Salivary Glands	Bulk RNA sequencing, DSP, mIHC	ACE2, B7-H3, BATF3, BDCA2, CCR5, CD206, CD3, CD39, CD56, CD8, CD80, CD83, FOXP3, Granzyme B, Ki-67, LAG3, PD-1, TIM-3	mIHC staining for SARS-CoV-2 NP

Abbreviations: ACE2: Angiotensin converting enzyme 2, CD: Cluster of differentiation, DSP: Digital spatial profiling, H&E: Haemotoxylin and eosin, Ig: Immunoglobulin, IHC: Immunohistochemistry, mIHC: Multiplex immunohistochemistry, NP: Nucleocapsid protein, RT-PCR: Real time-polymerase chain reaction, TEM: Transmission electron microscopy.

Supplementary Table 2. The clinical characteristics of all autopsy samples.

No	Age/Sex	Clinical Diagnosis	Cause(s) of Death	Time between death and anatomy(day)	Body Cavity Inspection	Multi-organ Failure (Y/N)						Comorbidity
						Pulmonary	Spleen	Liver	Kidney	Heart	Digestive tract	
Pt1	64Y/M	Secondary infection with COVID-19	RFRWS	1	B: PE (M), pale red; ascites (M),800-1000ml, pale red	Y	Y	N	N	Y	N	Multiple atherosclerotic plaques
Pt2	87Y/M	COVID-19 (Critical)	RFRWS; SARS-CoV-2 related pneumonia	1		Y	Y	N	N	Y	N	Microalveolar adenocarcinoma of the prostate gland; Atherosclerosis
Pt3	57Y/F	COVID-19	RFRWS; Secondary infection	1	B: PE (S), pale yellow; ascites (S), pale yellow	Y	Y	N	N	N	Y	Atherosclerotic plaques
Pt4	62Y/ M	COVID-19	RFRWS	1	B: PE (S-M), light yellow, slightly thick; No obvious ascites.	Y	Y	N	N	Y	N	
Pt5	73Y/F	COVID-19 (Severe pneumonia)	RFRWS; Secondary bacterial infection	1	B: PE (M), yellow; no obvious hydropericardium; ascites (S), pale yellow	Y	Y	Y	Y	N	Y	Coronary atherosclerosis
Pt6	88Y/M	COVID-19	RFRWS	0	B: PE (S), pale yellow	Y	N	N	N	Y	Y	Adenocarcinoma of prostate gland; Multiple atherosclerotic plaques
Pt7	76Y/M	COVID-19 (Critical)	RFRWS; Secondary bacterial infection	0	Hydropericardium (S), dark red; B: PE (M), dark red; ascites (S), dark red	Y	Y	Y	N	Y	Y	Prostatic hyperplasia; Aortosclerosis
Pt8	53Y/F	COVID-19	RFRWS	0	R: PE (S), pale yellow, 200ml Pulmonary adhered to the chest wall, especially middle and lower lobes (R) ascites (S), pale yellow	Y	Y	Y	Y	Y	Y	Prostatic hyperplasia; Aortosclerosis; microvascular transparent thrombosis
Pt9	74Y/F	COVID-19, Respiratory failure	Pulmonary thromboembolism; RFRWS	0	B: PE (S), pale yellow; ascites (S), pale yellow	Y	Y	N	N	Y	N	Multiple atherosclerotic plaques
Pt10	56Y/F	COVID-19	RFRWS; Pulmonary hyaline thromboembolism	0	B: PE (Ma), pale red; ascites (M), pale red	Y	Y	Y	N	Y	Y	Aortosclerosis
Pt11	84Y/F	COVID-19	RFRWS	0	B: PE (M), pale red; Pulmonary attached to the chest wall; ascites (M),800-1000ml, pale red	Y	Y	Y	Y	Y	Y	Atherosclerosis
Pt12	60Y/F	COVID-19	Hemorrhagic shock caused by dissecting aneurysm rupture; Multiple organ hemorrhage	0	B: PE (S), pale red; Massive hemorrhage and clot, about 11 * 5 cm in size	Y	Y	Y	N	Y	N	
Pt13	73Y/M	Lung squamous cell carcinoma										
Pt14	51Y/M	Pneumonia										
Pt15	64Y/M	Pulmonary bronchiectasis										
Pt16	66Y/F	COVID-19	RFRWS; Secondary bacterial infection	0	B: PE (M), pale yellow; ascites (M),800-1000ml, pale red	Y	N	N	Y	N	N	Multiple atherosclerotic plaques
Pt17	70Y/M	COVID-19 (Critical)	RFRWS; Multiple organ hemorrhage	0	B: PE (Ma), pale red; ascites (M), 800-1000ml, pale red	Y	Y	Y	Y	Y	Y	Atherosclerosis
Pt18	63Y/F	COVID-19	RFRWS; Pulmonary hemorrhage	1	B: PE (S), pale yellow; ascites (S), pale yellow	Y	Y	Y	Y	Y	Y	Aortosclerosis
Pt19	67Y/M	Viral pneumonia, hypertension, prostatic hyperplasia	RFRWS; Secondary infection	0	B: PE (Ma), pale red; ascites (Ma), 1000ml, pale red.	Y	Y	Y	Y	Y	Y	Prostatic hyperplasia; Atherosclerosis
Pt20	58Y/M	COVID-19	RFRWS	0	B: PE (S), pale yellow; ascites (S), pale yellow	Y	N	Y	N	N	Y	Atherosclerosis
Pt21	68Y/M	COVID-19	RP caused by pulmonary fungal infection; SARS-CoV-2 related	0	B: PE (S), pale yellow; No obvious ascites.	Y	Y	Y	N	Y	Y	Prostatic hyperplasia; Aortosclerosis; Endarteritis
Pt22	65Y/F	COVID-19 (Critical)	Heart failure related with dilated cardiomyopathy and infective endocarditis	0	B: PE (S), dark fawn, ascites (S), pale yellow	Y	Y	Y	N	Y	Y	Aortosclerosis; Endarteritis
Pt23	68Y/M		RFRWS; Multiple organ thromboembolism									
Pt24	80Y/F		RFRWS									
Pt25	59Y/M		RFRWS									

Abbreviation: PE: Pericardial effusion, RFRWS: Respiratory failure related with SARS-CoV-2, S: Small, M: Medium, Ma: Massive, B: Bilateral, L: Left, R: Right, M: Male, F: female, Pt: Patient, Y: Yes, N: No.

Supplementary Table 3. Antibodies used for mIHC, IHC, or DSP

Antibody	Source	Clone	Dilution	Catalogue number
ACE2	Abcam	EPR4435(2)	1:1500	ab108252
B7-H3	LSBio	SP206	1:50	LS-C210430
BATF3	Abcam	Polyclonal	1:150	ab981
BDCA2	Merck	10E6.1	1:25	MABF94
CCR5	R&D Systems	45523	2.5ug/ml	MAB181-100
CD206	Santa Cruz	D-1	1:200	MCA2155T
CD3	Dako	Polyclonal	1:200	A0452
CD39	Origene	OT12B10	1:800	TA804559
CD56	Leica Biosystems	CD564	1:200	NCL-L-CD56-504
CD8	Leica Biosystems	4B11	1:100	NCL-L-CD8-4B11
CD80	Abcam	EPR1157(2)	1:500	ab134120
CD83	Biolegend	HB15e	1:100	305302
FOXP3	Abcam	236A/E7	1:200	ab20034
GZMB	Leica Biosystems	GRB7	1:50	NCL-GRAN-B-L-CE
Ki-67	Dako	MIB-1	1:100	M7240
LAG3	CST	D2G40	1:800	#15372
PD-1	Cell Marque	NAT105	1:200	315M-96
SARS-CoV-2 (NP)	Novus Biologicals	Polyclonal	1:250	NB100-56576
TIM3	CST	D5D5R	1:400	#45208
Pan CK	Novus Biologicals	AE1/AE3	1:400	NBP2-33200DL488
CD68	Santa Cruz	KP1	1:100	sc-20060AF594
CD3	Origene	UMAB54	1:100	UM500048

Supplementary Table 4. Overview of the experimental use of all autopsy samples.

No	Lung	Liver	Heart	Kidney	Spleen	Lung	Liver	Heart	Kidney	Spleen	Lung	Liver	Heart	Kidney	Spleen
	**Bulk-RNA**	**Bulk-RNA**	**Bulk-RNA**	**Bulk-RNA**	**Bulk-RNA**	**DSP**	**DSP**	**DSP**	**DSP**	**DSP**	**mIHC**	**mIHC**	**mIHC**	**mIHC**	**mIHC**
Pt1	Y	N	N	N	N	Y	Y	N	N	Y	Y	Y	N	N	Y
Pt2	Y	Y	N	N	N	Y	N	N	Y	Y	Y	Y	N	Y	N
Pt3	Y	Y	N	Y	N	Y	N	N	N	N	Y	Y	N	Y	N
Pt4	Y	N	N	N	N	Y	Y	Y	N	N	Y	Y	Y	N	N
Pt5	Y	Y	Y	N	Y	Y	Y	Y	Y	N	Y	Y	Y	Y	N
Pt6	Y	N	Y	Y	Y	Y	Y	N	Y	Y	Y	Y	Y	Y	Y
Pt7	N	Y	N	N	N	Y	N	N	N	N	Y	Y	N	Y	N
Pt8	Y	N	N	N	N	N	N	N	N	N	Y	N	N	N	N
Pt9	Y	N	N	N	N	N	N	N	N	N	Y	N	N	N	N
Pt10	Y	N	N	N	N	N	N	N	N	N	Y	N	N	N	N
Pt11	Y	N	N	N	N	N	N	N	N	N	Y	N	N	N	N
Pt12	Y	N	N	N	N	N	N	N	N	N	N	N	N	N	N
Pt13	Y	N	N	N	N	N	N	N	N	N	N	N	N	N	N
Pt14	Y	N	N	N	N	N	N	N	N	N	N	N	N	N	N
Pt15	Y	N	N	N	N	N	N	N	N	N	N	N	N	N	N
Pt16	N	N	N	N	N	N	N	N	Y	Y	Y	N	N	Y	N
Pt17	N	N	N	N	N	N	N	N	N	N	Y	N	N	N	N
Pt18	N	N	N	N	N	N	N	N	N	N	Y	N	N	N	N
Pt19	N	N	N	N	N	N	N	N	N	N	Y	N	N	N	N
Pt20	N	N	N	N	N	N	N	N	N	N	Y	N	N	N	N
Pt21	N	N	N	N	N	N	N	N	N	N	Y	N	N	N	N
Pt22	N	N	N	N	N	N	N	N	N	N	Y	N	N	N	N
Pt23	N	N	N	N	N	N	N	N	N	N	Y	N	N	N	N
Pt24	N	N	N	N	N	N	N	N	N	N	Y	N	N	N	Y
Pt25	N	N	N	N	N	N	N	N	N	N	Y	N	N	N	Y

Abbreviation: Pt: Patient, Y: Yes, N: No, DSP: Digital Spatial Profiling, mIHC: multiplex immunohistochemistry.
