# Supplementary material for: Postmortem high-dimensional immune profiling of severe COVID-19 patients reveals distinct patterns of immunosuppression and immunoactivation
Source: Nat Commun. 2022 Jan 12;13:269. doi: 10.1038/s41467-021-27723-5 (PMC8755743; doi:10.1038/s41467-021-27723-5)
Supplement: Supplementary file 3 — Reporting Summary [file 41467_2021_27723_MOESM3_ESM.pdf]

nature research

Corresponding author(s): Cheng Sun

Last updated by author(s): Sep 1, 2021

Reporting Summary

Nature Research wishes to improve the reproducibility of the work that we publish. This form provides structure for consistency and transparency in reporting. For further information on Nature Research policies, see our Editorial Policies and the Editorial Policy Checklist.

Statistics

For all statistical analyses, confirm that the following items are present in the figure legend, table legend, main text, or Methods section.

n/a	Confirmed	
☐	☒	The exact sample size (n) for each experimental group/condition, given as a discrete number and unit of measurement
☐	☒	A statement on whether measurements were taken from distinct samples or whether the same sample was measured repeatedly
☐	☒	The statistical test(s) used AND whether they are one- or two-sided<br>*Only common tests should be described solely by name; describe more complex techniques in the Methods section.*
☒	☐	A description of all covariates tested
☐	☒	A description of any assumptions or corrections, such as tests of normality and adjustment for multiple comparisons
☐	☒	A full description of the statistical parameters including central tendency (e.g. means) or other basic estimates (e.g. regression coefficient) AND variation (e.g. standard deviation) or associated estimates of uncertainty (e.g. confidence intervals)
☐	☒	For null hypothesis testing, the test statistic (e.g. F, t, r) with confidence intervals, effect sizes, degrees of freedom and P value noted<br>*Give P values as exact values whenever suitable.*
☒	☐	For Bayesian analysis, information on the choice of priors and Markov chain Monte Carlo settings
☒	☐	For hierarchical and complex designs, identification of the appropriate level for tests and full reporting of outcomes
☒	☐	Estimates of effect sizes (e.g. Cohen's d, Pearson's r), indicating how they were calculated

Our web collection on statistics for biologists contains articles on many of the points above.

Software and code

Policy information about availability of computer code

Data collection	Raw FASTQ files were processed with Hisat2 software(v7.5.0) .QC of RNAseq data was performed using FastQC(v 0.11.9).The sequence reads were aligned to the human genome GRCh38, and aligned reads were counted within exons using FeatureCounts(v2.0.1).<br>The nCounter pipeline was used to collect oligonucleotide count data from the DSP GeoMX platform (NanoString Technologies, Inc.) under the Technology Access Program.<br>mIHC/IF was performed using an Opal Multiplex fIHC kit,images were acquired using a Vectra 3 pathology imaging system microscope (Akoya Biosciences).
Data analysis	All bulk-rna analysis carried out in R version 3.6.1. Versions of all packages used were as follows;<br>DESeq2(version 1.26.0),edgeR,(version 3.28.1),ggplot2(version 3.3.2),topGO(version 2.38.1),clusterProfiler (version 3.14.3) , pathview(version 1.26.0),AnnotationHub(version 2.18.0),org.Hs.eg.db(version 3.10.0),tidyverse(version 1.3.0),pheatmap(version 1.0.12),GSEABase(version 1.48.0),Rgraphviz(version 2.30.0),showtext(version 0.9),readr(version 1.3.1),stats(version 3.6.1),enrichplot(version1.6.1),RcolorBrewer(version 1.1-2),ggrepel(version 0.8.2)<br>GraphPad Prism(version 8.0.2) was used where listed for other statistical testing.<br>All mIHC analysis were carried out in Python v3.8.2. Versions of relevant packages used are as follows:<br>matplotlib (v3.2.1), numpy (v1.18.5), pandas (v1.0.5), scipy (v1.3.2), statsmodels (v0.11.1), inForm(v2.4.2)

For manuscripts utilizing custom algorithms or software that are central to the research but not yet described in published literature, software must be made available to editors and reviewers. We strongly encourage code deposition in a community repository (e.g. GitHub). See the Nature Research guidelines for submitting code & software for further information.

Data

Policy information about availability of data

All manuscripts must include a data availability statement. This statement should provide the following information, where applicable:

- Accession codes, unique identifiers, or web links for publicly available datasets
- A list of figures that have associated raw data
- A description of any restrictions on data availability

The RNA-Seq and GeoMx data generated in this study have been deposited in the Gene Expression Omnibus (GEO) under accession numbers GSE182917 (https://www.ncbi.nlm.nih.gov/geo/query/acc.cgi?acc=GSE182917), GSE182920 (https://www.ncbi.nlm.nih.gov/geo/query/acc.cgi?acc=GSE182920). The images can be queried in the database BioStudies (https://www.ebi.ac.uk/biostudies/studies/S-BIAD170?query=S-BIAD170). Source data are provided with this paper.

Field-specific reporting

Please select the one below that is the best fit for your research. If you are not sure, read the appropriate sections before making your selection.

☒ Life sciences ☐ Behavioural & social sciences ☐ Ecological, evolutionary & environmental sciences

For a reference copy of the document with all sections, see nature.com/documents/nr-reporting-summary-flat.pdf

Life sciences study design

All studies must disclose on these points even when the disclosure is negative.

Sample size	We have collected all 22 patients with severe COVID-19 who had undergone autopsy that were available in the Huoshenshan Hospital in the period of the study. No sample size calculation was performed. Sample size is chosen based on the standard of the corresponding field. The age of the patients ranged from 51 to 88 years, with a median age of 66 years. The three age- and sex-matched lung controls was used in the bulk RNA-sequencing analysis were obtained from patients suffering from lung squamous cell carcinoma, pneumonia, and pulmonary bronchiectasis, respectively. The age of these patients ranged from 51 to 73 years, with a median age of 64.0 years.
Data exclusions	no data exclusions apply.
Replication	Experiments were successfully repeated at least three times on independent samples.
Randomization	This study is on autopsy material from patients who succumbed to SARS-CoV-2 infection. Control samples were not randomized but selected based on matching age and gender to SARS-CoV-2 RT-PCR confirmed infected subjects samples age and gender. So, there was no need of randomization.
Blinding	This study is on autopsy material from patients who succumbed to SARS-CoV-2 infection. We were not blinded to subjects as recruitment was based on RT-PCR confirmed COVID-19 infection, and control samples selected based on age and gender matching.

Reporting for specific materials, systems and methods

We require information from authors about some types of materials, experimental systems and methods used in many studies. Here, indicate whether each material, system or method listed is relevant to your study. If you are not sure if a list item applies to your research, read the appropriate section before selecting a response.

Materials & experimental systems

n/a	Involved in the study	
☐	☒	Antibodies
☒	☐	Eukaryotic cell lines
☒	☐	Palaeontology and archaeology
☒	☐	Animals and other organisms
☐	☒	Human research participants
☒	☐	Clinical data
☒	☐	Dual use research of concern

Methods

n/a	Involved in the study	
☒	☐	ChIP-seq
☒	☐	Flow cytometry
☒	☐	MRI-based neuroimaging

Antibodies

Antibodies used

Antibody Source Clone Dilution Catalogue number
ACE2 Abcam EPR4435(2) 1:1500 ab108252
B7-H3 LSBio SP206 1:50 LS-C210430
BATF3 Abcam Polyclonal 1:150 ab981
BDCA2 Merck 10E6.1 1:25 MABF94

CCR5 R&D Systems 45523 2.5ug/ml MAB181-100
CD206 Santa Cruz D-1 1:200 MCA2155T
CD3 Dako Polyclonal 1:200 A0452
CD39 Origene OT12B10 1:800 TA804559
CD56 Leica Biosystems CD564 1:200 NCL-L-CD56-504
CD8 Leica Biosystems 4B11 1:100 NCL-L-CD8-4B11
CD80 Abcam EPR1157(2) 1:500 ab134120
CD83 Biolegend HB15e 1:100 305302
FOXP3 Abcam 236A/E7 1:200 ab20034
GZMB Leica Biosystems GRB7 1:50 NCL-GRAN-B-L-CE
Ki-67 Dako MIB-1 1:100 M7240
LAG3 CST D2G40 1:800 #15372
PD-1 Cell Marque NAT105 1:200 315M-96
SARS-CoV-2 (NP) Novus Biologicals Polyclonal 1:250 NB100-56576
TIM3 CST D5D5R 1:400 #45208
Pan CK Novus Biologicals AE1/AE3 1:400 NBP2-33200DL488
CD68 Santa Cruz KP1 1:100 sc-20060AF594
CD3 Origene UMAB54 1:100 UM500048
Antibodies used for Multiplex Immunohistochemistry/Immunofluorescence are also as listed in Supplementary Table 3

Validation

All antibodies were validated according to respective manufacturer's information and citations. All antibodies were used in the field of human research and all antibodies were titrated by us prior to use on patient samples. Commercial primary antibodies were validated by the manufacturers and validation statements are available on the manufacturer's website. Product specification sheets for each antibody describe from the company the expected cell proportion based on cell staining.

Human research participants

Policy information about studies involving human research participants

Population characteristics

Characteristics of the patients in this study are fully described in the supply table2. The age of patients ranged from 51 to 88, and included 14 male and 11 female patients. 22 patients in this study died of SARS-CoV-2-associated respiratory failure, and autopsies were carried out at the anatomy mobile cabin of Vulcan mountain .

Recruitment

This study is on autopsy material from patients who succumbed to SARS-CoV-2 infection. All the tissues of each viscera were obtained from each patient after COVID-19 infection. The patients in this study donated their remains voluntarily. There was no bias to the patient recruitment or tissue selection.

Ethics oversight

This project is approved by research permit for project KY2020027 of Ethics Committee of the First Affiliated Hospital of Army Medical University,PLA .Written informed consent was obtained from all subjects.

Note that full information on the approval of the study protocol must also be provided in the manuscript.
